# miR-506-3p induces autophagy of renal tubular epithelial cells in sepsis through targeting PI3K pathway

**DOI:** 10.18632/aging.204759

**Published:** 2023-06-02

**Authors:** Yun Dong, Xiaorui Han, Yong Yang, Hui Shi

**Affiliations:** 1 Department of Critical Care Medicine, Xingtai People’s Hospital, Xingtai 054000, Hebei, China; 2 Department of Gynaecology and Obstetrics, Xingtai People’s Hospital, Xingtai 054000, Hebei, China; 3 Department of Traumatic Orthopedics, Xingtai People’s Hospital, Xingtai 054000, Hebei, China

**Keywords:** miR-506-3p, sepsis-associated acute kidney injury, autophagy, PI3K pathway, renal tubular epithelial cells

## Abstract

Objective: To explore the effect of micro ribonucleic acid (miR)-506-3p on autophagy of renal tubular epithelial cells in sepsis and its mechanism.

Methods: It was found through bioinformatics analysis that phosphatidylinositol 3-kinase catalytic subunit alpha (PIK3CA) was expressed at a low level in sepsis, and miR-506-3p had a targeted regulatory effect on PIK3CA. 40 8-week-old male C57BL/6 mice were randomly divided into control miR-506-3p NC group, control miR-506-3p OE group, sepsis miR-506-3p NC group, sepsis miR-506-3p OE group and sepsis miR-506-3p KD group. The pathological changes in kidney tissues of mice in each group were observed by hematoxylin-eosin (HE) staining and TUNEL staining, and mitochondria and autophagosomes were visualized by transmission electron microscopy. CCK8 assay was performed to detect the effect of miR-506-3p on the proliferation capacity of renal tubular epithelial cells. The changes in the expression of PI3K-Akt pathway proteins, mTOR and autophagy proteins were tested by Western blotting.

Results: The injury and apoptotic positive cells were suppressed and decreased in miR-506-3p OE mice vs. NC group. miR-506-3p could increase the number of mitochondria and autophagosomes in kidney tissues. After introduction of exogenous miR-506-3p OE into renal tubular epithelial cells, the expressions of PI3K pathway proteins were significantly inhibited, while the expressions of autophagy proteins were significantly enhanced. After 740Y-P was added, the expressions of associated proteins had no significant changes in each group.

Conclusion: Overexpression of miR-506-3p can enhance the autophagy of renal tubular epithelial cells in sepsis through inhibiting the PI3K signaling pathway.

## INTRODUCTION

Sepsis-associated acute kidney injury (SA-AKI) is the most common type of AKI in the intensive care unit [1]. Early anti-infective therapy, fluid resuscitation, vasoactive drugs and organ support therapy have been the dominant therapies for SA-AKI [2], but they have poor effects. It has been found that SA-AKI is accompanied by autophagy changes, which have a certain impact on the disease [3]. Autophagy plays a dual role in SA-AKI induced by lipopolysaccharide (LPS) or cecal ligation and puncture [4], which promotes the survival of cells and tissues in the early stage and exerts an injury effect in the late stage [5].

It was found through Gene Expression Omnibus (GEO) database that the expression of phosphatidylinositol 3-kinase catalytic subunit alpha (PIK3CA) was down-regulated in sepsis compared with that in normal samples. The upstream micro ribonucleic acids (miRNAs) of PIK3CA were evaluated through miRDB (http://mirdb.org/miRDB/), starBase (http://starbase.sysu.edu.cn) and TargetScan (http://www.targetscan.org/vert_72/). The results showed that miR-506-3p had a targeted regulatory effect on PIK3CA. It has been confirmed that miRNAs, a class of small regulatory RNA, are important players in the development of inflammation, cell proliferation, apoptosis and autophagy. miR-506-3p possesses a variety of biological functions in cells, and it is implicated in regulating the inflammatory response and autophagy [6]. At the same time, studies have shown that the phosphatidylinositol 3-kinase (PI3K) pathway is of importance in the pathogenesis of diseases [7], and involved in cell differentiation, proliferation, autophagy and apoptosis [8]. However, the mechanisms of miR-506 and PI3K-Akt signaling pathways in autophagy in SA-AKI have not been reported yet. In the above context, it is speculated that miR-506-3p may affect the autophagy of renal tubular epithelial cells in sepsis by targeting the PI3K pathway.

In the present study, gene screening, enrichment analysis and pathway analysis were performed by bioinformatics, the effect of miR-506-3p on autophagy of renal tubular epithelial cells in sepsis and its mechanism were detected through *in vivo* and *in vitro* experiments, and the possible therapeutic target for SA-AKI was explored.

## METHODS

### Bioinformatics analysis

#### Dataset acquisition and preprocessing

The datasets (GSE101639 and GSE154918) related to sepsis-related gene expression were searched and downloaded using the data analysis tool GEO2R (https://www.ncbi.nlm.nih.gov/geo/geo2r/). The gene expression in GSE101639 was subjected to quantile normalization.

#### Screening of differentially expressed genes (DEGs)

The DEGs were screened (|logFC|>2 and *p* < 0.05) in normal group and sepsis group. Enrichment analysis was conducted using the online tool DAVID and R/Bioconductor package. The differential expression of miRNAs in GSE101639 was analyzed by the Bayesian method.

#### Potential pathways of DEGs and biological process analysis

The upstream miRNAs of PIK3CA were predicted through intersection of DEGs using miRDB, starBase and TargetScan. The functional and pathway enrichment was checked by DAVID Knowledgebase (https://david.ncifcrf.gov/). The enriched GO terms or KEGG pathway annotations were obtained by the R package, and the key genes were obtained by Cytoscape and cytoHubba.

### Laboratory reagents

LPS (10 mg/kg) was purchased from Sigma (USA), the PIK3CA (ab40776, 1:1000, Abcam), p-PI3K (ab278545, 1:2000, Abcam), p-mTOR (ab109268, 1:1000, Abcam), Beclin (ab207612, 1:2000, Abcam), LC-3II (ab192890, 1:2000, Abcam) and ATG5 (ab108327, 1:1000, Abcam) antibodies were purchased from Abcam (USA), miR-506-3p OE NC and miR-506-3p OE (50 nM per well, RIBOBIO) were synthesized by Sangon, terminal deoxynucleotidyl transferase-mediated dUTP nick end labeling (TUNEL) kits were purchased from Wuhan Boster Biological Technology Co., Ltd., and Western blotting reagents were purchased from Beijing Solarbio Science and Technology Co., Ltd.

### Animal modeling and grouping

A total of 40 8-week-old male C57BL/6 mice were randomly divided into control group (miR-506-3p OE NC group, *n* = 10), miR-506-3p overexpression group (miR-506-3p OE group, *n* = 10), sepsis model control group (sepsis miR-506-3p OE NC group, *n* = 10) and sepsis model miR-506-3p overexpression group (sepsis miR-506-3p OE group, *n* = 10). In sepsis miR-506-3p OE NC group, sepsis miR-506-3p OE group and sepsis miR-506-3p KD group. LPS was intraperitoneally injected into mice to induce SA-AKI modeling. 24 h later, the mice were sacrificed, and the kidney tissues were harvested for later experiments.

### Observation of pathological changes in kidney tissues by hematoxylin-eosin (HE) staining

The kidney tissues harvested were immediately fixed in paraformaldehyde solution, dehydrated with gradient alcohol, rinsed with tap water 3 times (5 min/time), stained with hematoxylin for 5 min, rinsed again with running water, and differentiated with 5% acetic acid for 1 min, after which the tissues turned pale blue, followed by eosin staining for 1 min, rinsing with running water, and dehydration with gradient alcohol for 10 s and xylene for 1 min. Then the tissues were air-dried in a fume cupboard, and mounted with neutral balsam.

### Detection of apoptosis of renal tubular epithelial cells in kidney tissues by TUNEL staining

The kidney tissues were fixed with 10% neutral formalin, embedded in paraffin and sliced into sections, and the mode of cell death was detected according to the instructions of TUNEL apoptosis detection kits. The images were observed, collected and analyzed under a fluorescence microscope.

### Electron microscopy

The samples were fixed overnight in 0.1 M sodium cacodylate buffer in 2.5% glutaraldehyde, post-fixed in 1% osmium tetroxide in 0.1 M sodium cacodylate buffer, dehydrated in gradient ethanol and propylene oxide, and embedded in EPON-812 resin (Sigma-Aldrich, 45345). The semi-thin cross-section was stained with 0.5% water-based toluidine blue O (Sigma-Aldrich, 89640), and bright-field images were acquired using a ZEISS Axioskop equipped with an RT slider point camera. The ultrathin transverse section was stained with uranyl acetate and lead citrate, and images were acquired by the JEM-1400Flash electron microscope. These images were imported into the Image-Pro Plus software for morphometric area measurement in EM.

### Cell transfection and culture

The renal tubular epithelial cells were routinely cultured with DMEM containing 10% fetal bovine serum in a 5% CO_2_ incubator at 37°C, and the medium was replaced every 2–3 d, followed by passage at 1:3. The renal tubular epithelial cells in the logarithmic growth phase were harvested and inoculated into a 6-well plate at an appropriate density. Upon reaching 80% confluence, the cells were transfected with miR-506-3p OE NC and miR-506-3p OE (miR-506-3p NC group and miR-506-3p OE group) according to the instructions of Lipofectamine2000, and collected 48 h later. Then 740Y-P (PI3K agonist) was added into each group to set up miR-506-3p NC+740Y-P group and miR-506-3p OE+740Y-P group for later experiments.

### CCK8 assay for proliferation capacity

When the tubular epithelial cells of the miR-506-3p NC group and the miR-506-3p OE group were cultured till a density of 80–90%, the medium was discarded, and the cells were washed twice with an appropriate amount of PBS and added with 1–2 mL of pancreatic enzyme containing 0.25% EDTA each cell. Then the appropriate length of digestion was selected, 2 mL of low-glucose DMEM was added to terminate digestion and the supernatant was discarded after centrifugation. After an appropriate amount of low-glucose DMEM was aspirated, the cells were suspended and viable cells were counted. The cells were then seeded into 96-well plates (200 μL each well), and incubated in a 5% CO_2_ incubator at 37°C. After 12 h of pre-incubation, 10 μL of CCK8 solution was added to each well, and the cells were incubated in the incubator for another 2 h. Finally, the OD value was measured at 450 nm.

### Transmission electron microscopy

The kidney was harvested from each newborn mouse, washed with PBS, placed on an ice box, aspirated with a disposable dropper, and fixed with 2.5% glutaraldehyde solution. After rinsing with 0.1 mol·L^−1^ phosphate buffer, the sample was transferred to and fixed with 1% osmium acid at 4°C for 3 h. After rinsing with 0.1 mol·L^−1^ phosphate buffer, the sample was dehydrated with gradient ethanol: 30% ethanol for 15 min, 50% ethanol for 15 min, 70 ethanol uranyl acetate overnight, 80% ethanol for 15 min, and 90% ethanol: 90% acetone (1:1) for 15 min (all steps were performed at 4°C), followed by treatment with 100% acetone 15 min × 3 h (room temperature), pure acetone + embedding solution (1:1) 2 h (room temperature), and pure embedded solution in a 37°C oven for 3 h. Then the sample was embedded in the embedding solution in a 37°C oven for 12 h and a pure embedding solution oven at 60°C for 48 h. The semi-thin sections with a thickness of 1 μm were stained with azure blue and observed under a light microscope to determine the position of the retina and the structure of 10 layers. The ultrathin sections at a thickness of 80 nm were fished out and placed on the supporting membrane copper mesh, followed by lead citrate staining for 15 min, washing with distilled water 3 times, natural drying, and transmission electron microscopy.

### Detection of miR-506-3p expression in renal tubular epithelial cells by real-time fluorescence quantitative PCR

The total RNA was extracted from the renal tubular epithelial cells in kidney tissues, and its concentration was measured using a spectrophotometer. The RNA extracted was reversely transcribed into cDNA according to the kit instructions, and subjected to real-time fluorescence quantitative PCR. The reaction system (20 μL) was composed of 10 μL of nucleic acid amplification reaction solution, 2 μL of primer probe reaction solution A/B, 6 μL of nuclease-free water, and 2 μL of DNA samples as the reaction template. The amplification was conducted under the following conditions: 95°C for 5 min × 1 cycle, (95°C for 15 s, 60°C for 1 min) × 40 cycles, with 3 replicates for each in each group.

### Detection of expressions of PIK3CA and autophagy protein Beclin by immunohistochemistry

The kidney tissues were embedded in paraffin, sliced into sections, and deparaffinized, followed by antigen retrieval. The sections were added dropwise with 3% hydrogen peroxide to block endogenous peroxidase, incubated at room temperature for 15 min, rinsed with PBS 3 times (3 min/time), blocked with serum and incubated with PIK3CA and Beclin primary antibodies and HRP-labeled secondary antibodies, followed by color development with DAB and hematoxylin counterstaining. After dehydration and mounting, the sections were photographed and analyzed under an electron microscope.

### Detection of expressions of PI3K pathway proteins PIK3CA, p-PI3K and p-mTOR and autophagy proteins Beclin, LC-3II and ATG5 by Western blotting

The protein was extracted from the renal tubular epithelial cells in kidney tissues, and its concentration was determined by BCA method. After the protein was separated by SDS-PAGE, the target protein was transferred onto a PVDF membrane, blocked at room temperature for 2 h, and incubated with the corresponding primary antibodies of PIK3CA, p-PI3K, p-mTOR, Beclin, LC-3II and ATG5 (1:1,000) at 4°C overnight. The next day, the excess primary antibodies were washed away, and the corresponding secondary antibodies (1:5,000) were added to incubate the protein at room temperature for 2 h. After the excess secondary antibodies were washed away, the chemiluminescence solution was dropwise added, the images were acquired using gel imaging system, and the gray value was analyzed using BandScan.

### Statistical analysis

SPSS 19.0 software was used for data processing. Experimental data were expressed as mean ± standard deviation (mean ± SD) in data processing and statistical analysis. The mean was compared among groups by one-way analysis of variance, and pairwise comparison was made by SNK test. *p* < 0.05 was considered to be statistically significant.

## RESULTS

### Bioinformatics analysis

#### Dataset acquisition

**Figure 1:**
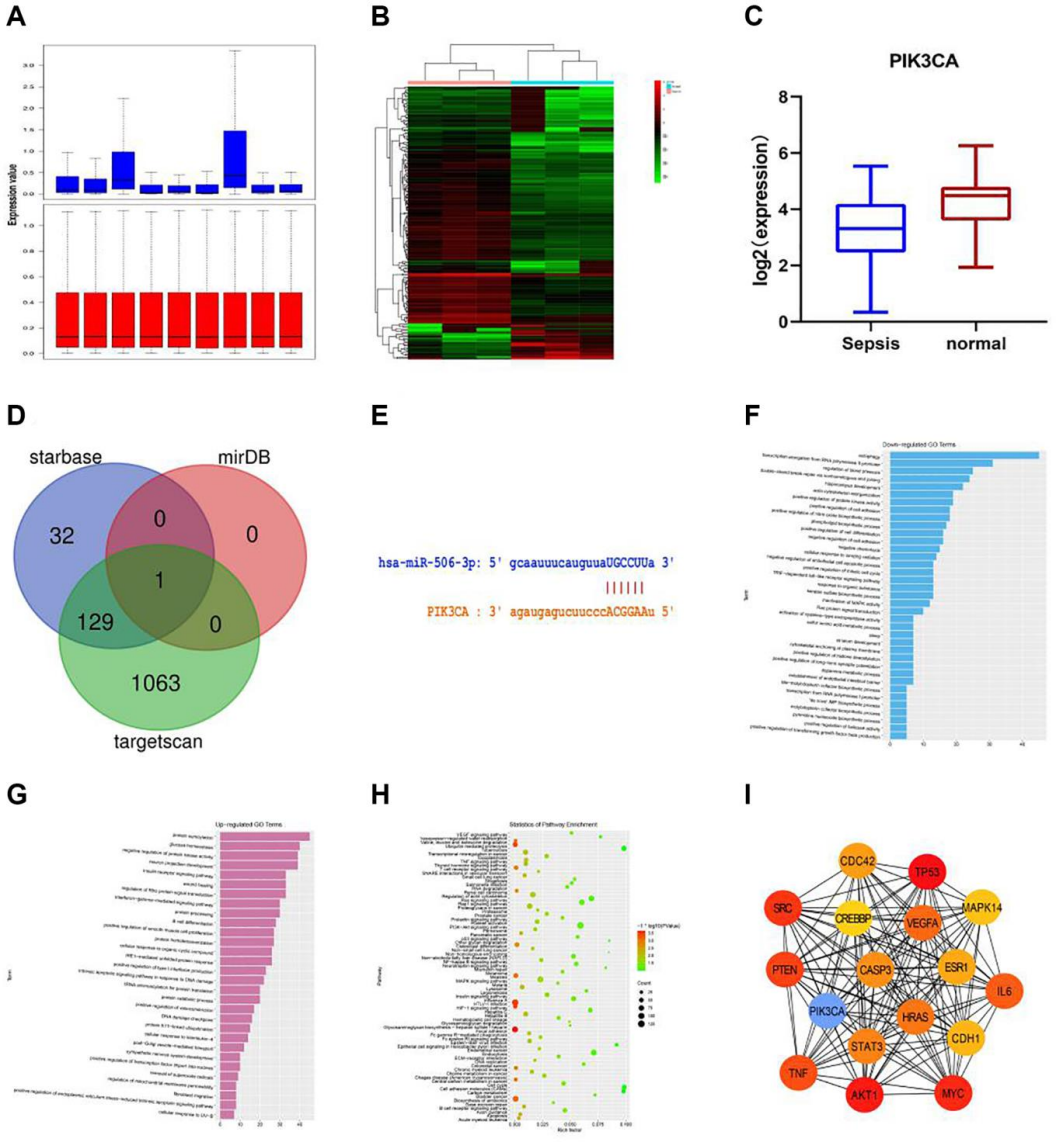
**Bioinformatics analysis.** (**A**) The gene expression in GSE101639 was subjected to quantile normalization. (**B**) DEGs in normal group and sepsis group. (**C**) It was found in GSE154918 that the expression of PIK3CA was down-regulated in sepsis compared with that in normal samples. (**D**) The upstream miRNAs of PIK3CA were evaluated through miRDB, starBase and TargetScan, and the DEG of miR-506, a key regulator of PIK3CA, was obtained from the intersection. (**E**) miR-506 of PIK3CA had a targeted binding region. (**F**) GO enrichment analysis was performed using the online tool DAVID. A total of 37 down-regulated pathways were enriched, including autophagy, transcription elongation from RNA polymerase II promoter and regulation of blood pressure. (**G**) 30 up-regulated pathways, including protein SUMOylation, glucose homeostasis and negative regulation of protein kinase activity. (**H**) KEGG pathway analysis was conducted on the integrated DEGs using DAVID. (**I**) The hub gene was obtained by Cytoscape.

The datasets (GSE101639 and GSE154918) related to sepsis-related gene expression were searched and downloaded using the data analysis tool GEO2R (https://www.ncbi.nlm.nih.gov/geo/geo2r/). The gene expression in GSE101639 was subjected to quantile normalization, and the results before and after normalization are shown in Figure 1A.

#### Screening of DEGs

Paired-samples *t*-test was performed (|logFC|>2 and *p* < 0.05) to evaluate DEGs between normal group and sepsis group (Figure 1B). It was found in GSE154918 that the expression of PIK3CA was down-regulated in sepsis compared with that in normal samples (Figure 1C). The upstream miRNAs of PIK3CA were evaluated through miRDB, starBase and TargetScan, and the DEG of miR-506, a key regulator of PIK3CA, was obtained from the intersection (Figure 1D).

#### Potential pathways of DEGs and biological process analysis

It was found through miRDB (http://mirdb.org/miRDB/) and starBase (http://starbase.sysu.edu.cn) that miR-506 of PIK3CA had a targeted binding region (Figure 1E). The role of PIK3CA in sepsis was further studied by RNA sequence analysis of shPIK3CA-transfected plasma samples. In addition, GO enrichment analysis was performed using the online tool DAVID. A total of 37 down-regulated pathways were enriched, including autophagy, transcription elongation from RNA polymerase II promoter and regulation of blood pressure (Figure 1F), and 30 up-regulated pathways were also identified, including protein SUMOylation, glucose homeostasis and negative regulation of protein kinase activity (Figure 1G). Besides, KEGG pathway analysis was conducted on the integrated DEGs using DAVID, including PI3K-Akt signaling pathway, HTLV-I infection and MAPK signaling pathway (Figure 1H), and the hub gene was obtained by Cytoscape. Therefore, PIK3CA was considered as a key gene involved in the negative regulation of autophagy cascade (Figure 1I).

### Effect of overexpression of miR-506-3p on pathological changes in kidney tissues in SA-AKI

**Figure 2:**
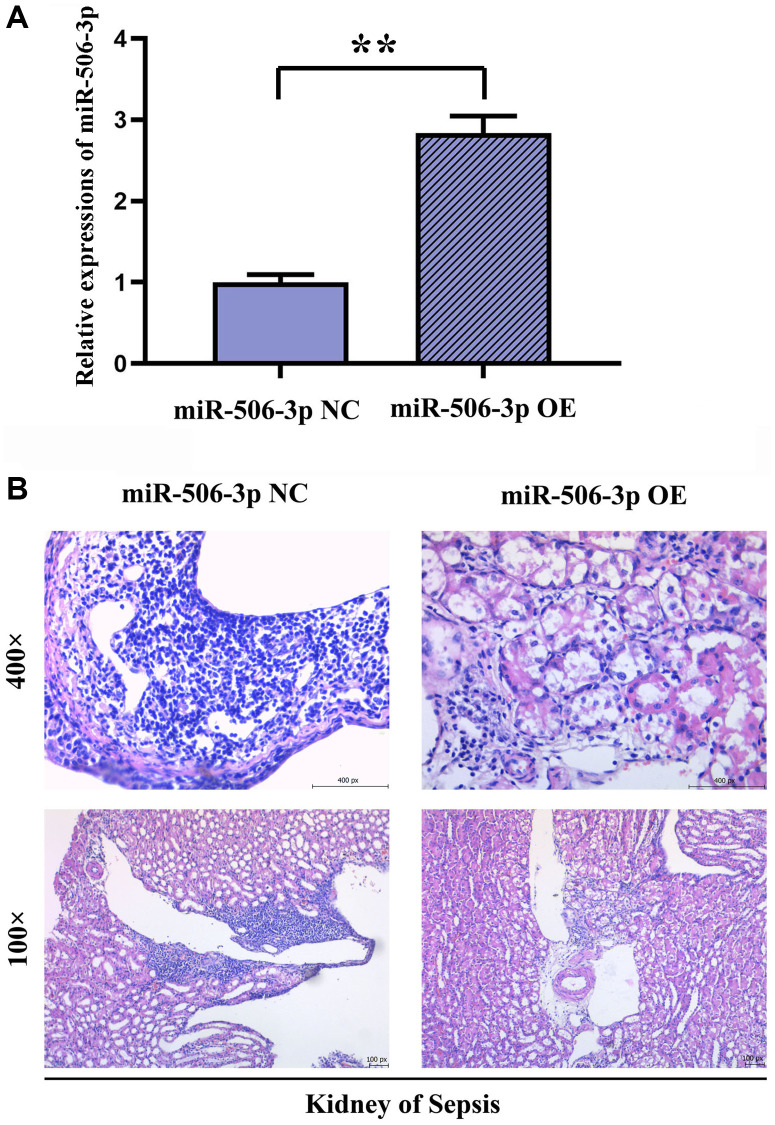
**Effect of overexpression of miR-506-3p on pathological changes in kidney tissues in SA-AKI.** (**A**) Expression of miR-506-3p in each group. (**B**) Through HE staining, it was found that the degree of tissue damage in the miR-506-3p OE group was significantly weakened compared with that in the miR-506-3p NC group.

The successful miR-506-3p transfection was detected by real-time fluorescence quantitative PCR. After HE staining, it was observed under the electron microscope that there were no significant changes in the pathology of kidney tissues, autophagy of renal tubular epithelial cells and mitochondrial morphology in miR-506-3p OE NC group, miR-506-3p OE group and sepsis miR-506-3p OE NC group. Compared with those in sepsis miR-506-3p OE NC group, the kidney tissue damage, mitochondrial morphological changes and apoptosis were significantly alleviated, while the number of autophagosomes in renal tubular epithelial cells was significantly increased in sepsis miR-506-3p OE group (Figure 2).

### Overexpression of miR-506-3p could reduce apoptosis after SA-AKI

**Figure 3:**
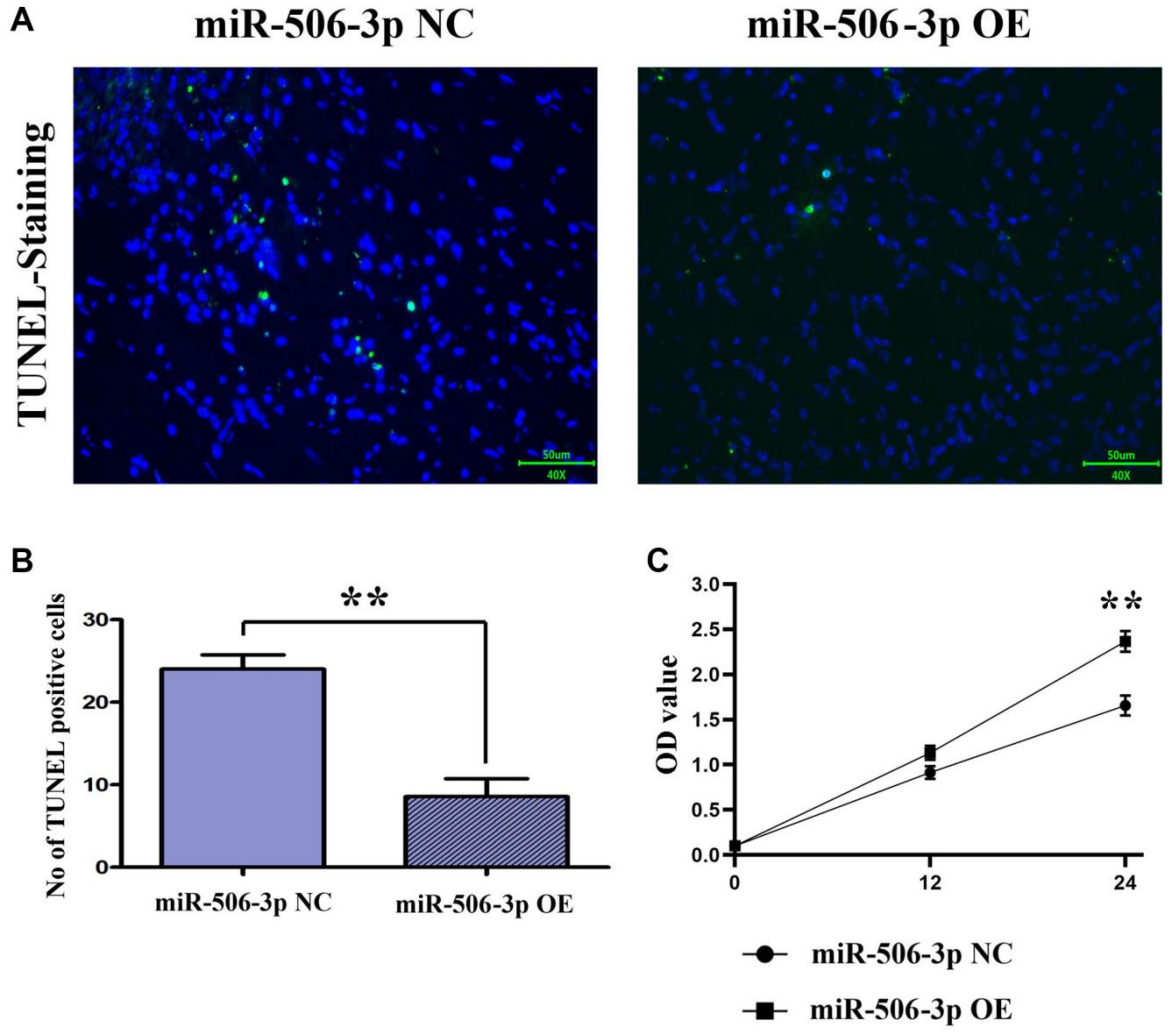
**Overexpression of miR-506-3p could reduce apoptosis and promote proliferation after SA-AKI.** (**A**, **B**) The results of TUNEL staining assay showed that apoptosis was significantly weakened in the miR-506-3p OE group compared to that in the miR-506-3p NC group. (**C**) Through CCK8 assay, it was found that the cell proliferative capacity of the miR-506-3p OE group was significantly enhanced compared to that in the miR-506-3p NC group.

The apoptosis of renal tubular epithelial cells in kidney tissues was detected by TUNEL staining. The results showed that the apoptosis in kidney tissues was significantly weakened in sepsis miR-506-3p OE group compared with that in sepsis miR-506-3p OE NC group (*p* < 0.01), suggesting that overexpression of miR-506-3p can effectively reduce apoptosis in kidney tissues after SA-AKI (Figure 3A, 3B).

### Overexpression of miR-506-3p could reduce the proliferative capacity

The proliferation capacity of cells was detected by CCK8 assay. After 24 h of culture, it was found that the proliferation capacity of renal tubular epithelial cells in the miR-506-3p NC group was significantly stronger than that in the miR-506-3p OE group (Figure 3C).

### Expression of miR-506-3p significantly rose in renal tubular epithelial cells overexpressing miR-506-3p

**Figure 4:**
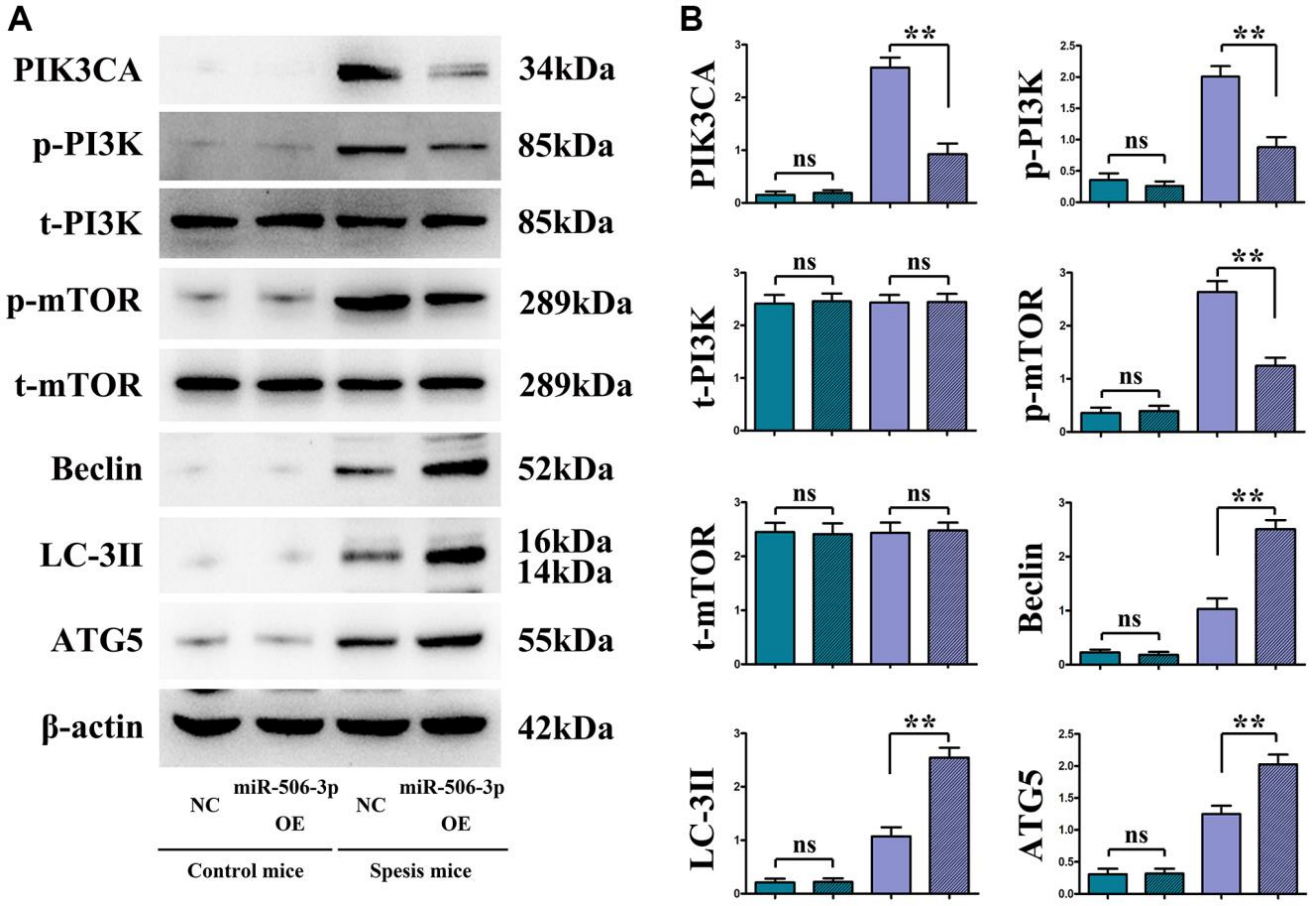
**Overexpressed miR-506-3p inhibited the expression of PI3K pathway-related proteins and enhanced the expression of related autophagy proteins.** (**A**) Protein bands of PIK3CA, p-PI3K, t-PI3K, p-mTOR, t-mTOR, Beclin, LC-3II and ATG5. (**B**) Relative protein expressions of PIK3CA, p-PI3K, t-PI3K, p-mTOR, t-mTOR, Beclin, LC-3II and ATG5. ^**^P < 0.01, ^ns^*P* > 0.05.

The mRNA expression of miR-506-3p in each group was detected by fluorescence quantitative PCR. The results revealed that the expression of miR-506-3p in renal tubular epithelial cells was significantly higher in miR-506-3p OE groups than that in miR-506-3p OE NC groups (*p* < 0.01) (Figure 4).

### Overexpression of miR-506-3p decreased the expressions of PI3K signaling pathway proteins PIK3CA, PI3K and mTOR, but increased the expressions of autophagy proteins Beclin, LC-3II and ATG5

**Figure 5:**
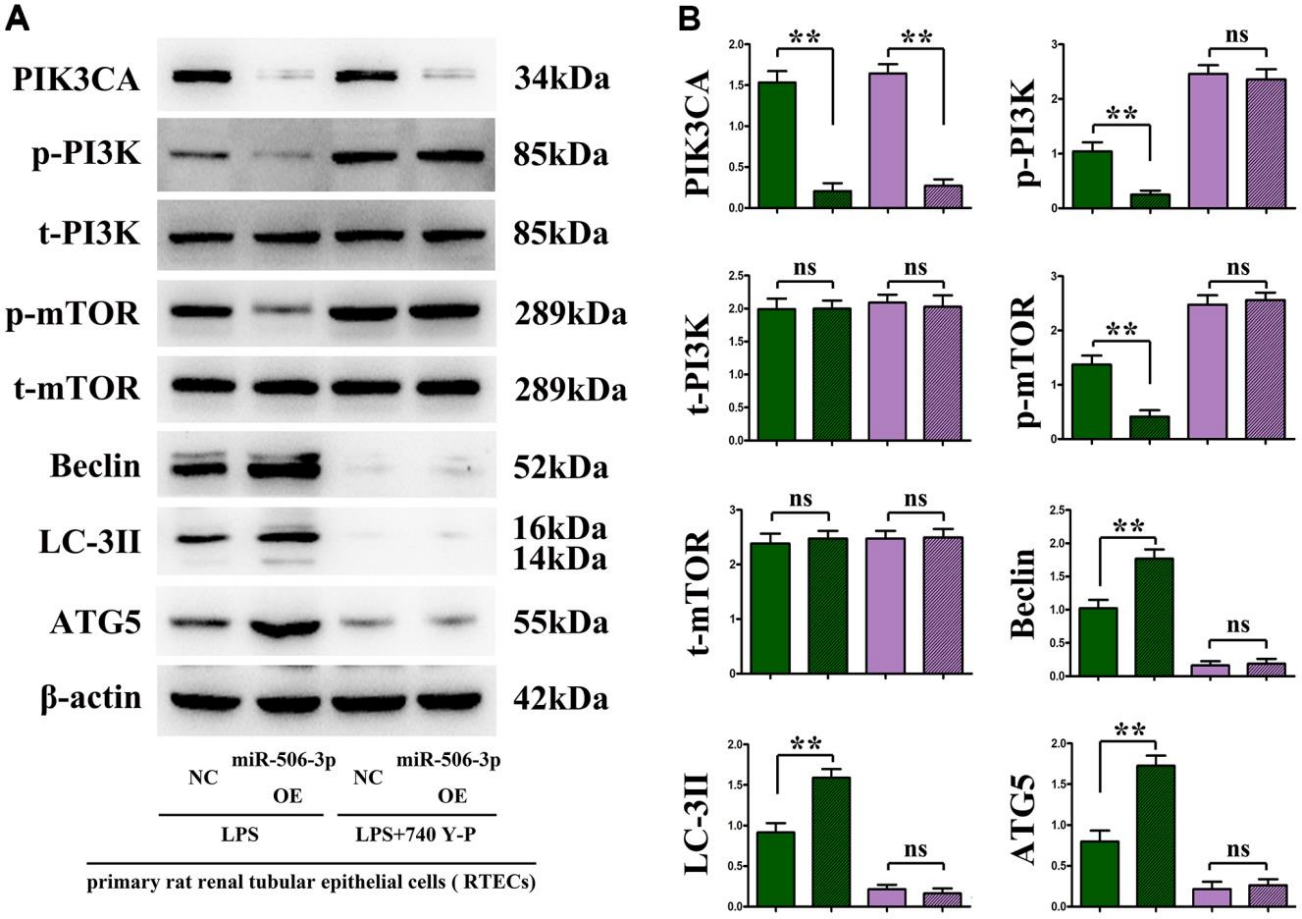
***In vitro* experiments demonstrated that the overexpression of miR-506-3p could enhance autophagy by regulating the PI3K signaling pathway.** (**A**) Protein bands of PIK3CA, p-PI3K, t-PI3K, p-mTOR, t-mTOR, Beclin, LC-3II and ATG5. (**B**) Relative protein expressions of PIK3CA, p-PI3K, t-PI3K, p-mTOR, t-mTOR, Beclin, LC-3II and ATG5. ^**^P < 0.01, ^ns^*P* > 0.05.

The changes in the expressions of PI3K-Akt pathway proteins (PIK3CA, p-PI3K, and p-mTOR) and autophagy proteins (Beclin, LC-3II, and ATG5) were detected through immunohistochemistry and Western blotting. The results showed that sepsis miR-506-3p OE group had significantly decreased expressions of PIK3CA, PI3K and mTOR (*p* < 0.01), and significantly increased expressions of Beclin, LC-3II and ATG5 compared with sepsis miR-506-3p OE NC group (*p* < 0.01). After introduction of exogenous miR-506-3p OE into renal tubular epithelial cells, the expressions of PIK3CA, p-PI3K, and p-mTOR were significantly inhibited, while the expressions of Beclin, LC-3II, and ATG5 were significantly enhanced (*p* < 0.01). After 740Y-P was added, the expressions of p-PI3K, p-mTOR, Beclin, LC-3II and ATG5 had no significant changes, but the expression of PIK3CA declined in miR-506-3p OE+740Y-P group compared with those in miR-506-3p NC+740Y-P group (*p* < 0.01). The above results demonstrate that exogenous overexpression of miR-506-3p can regulate the expression of autophagy- related proteins through inhibiting the PI3K pathway, thereby enhancing the autophagy of renal tubular epithelial cells in sepsis (Figure 5).

### Overexpression of miR-506-3p could promote autophagy

**Figure 6:**
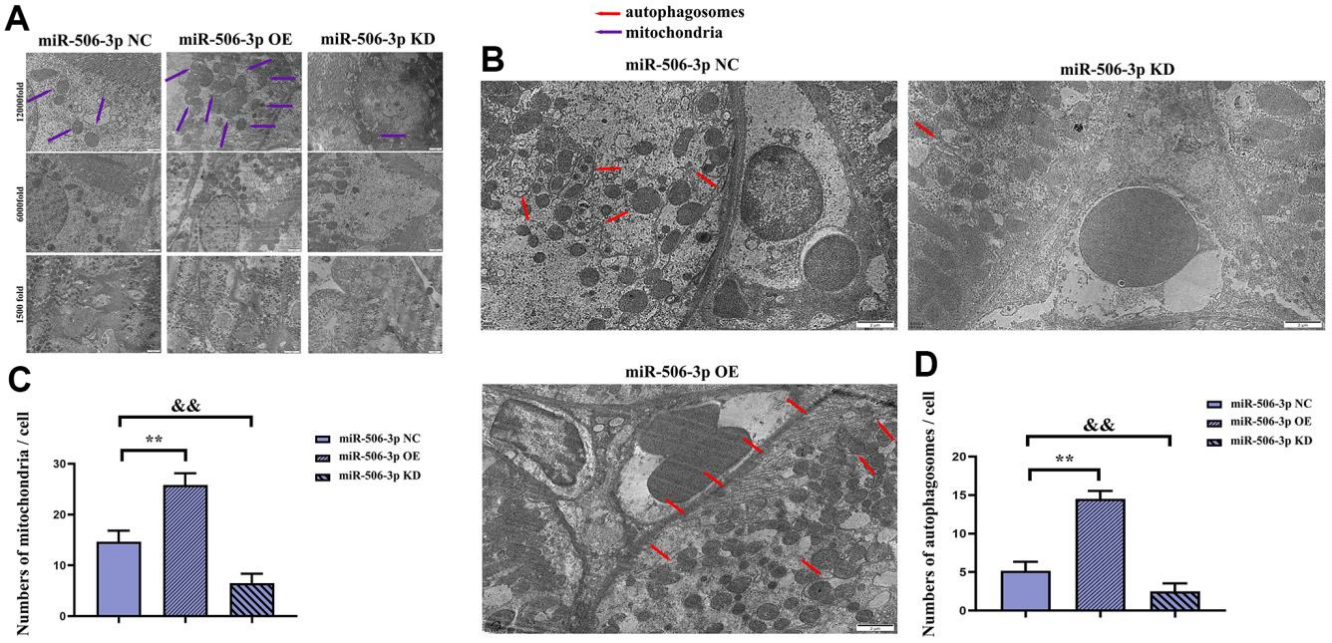
**Effect of miR-506-3p on autophagy.** (**A**) Mitochondria in kidney tissues (1500x, 6000x, and 12000x). (**B**) Autophagosomes in kidney tissues (1500x, 6000x, and 12000x). (**C**) Statistics on the number of mitochondria in each cell. (**D**) Statistics on the number of autophagosomes in each cell. ^**^P < 0.01, ^&&^*P* < 0.01.

**Figure 7:**
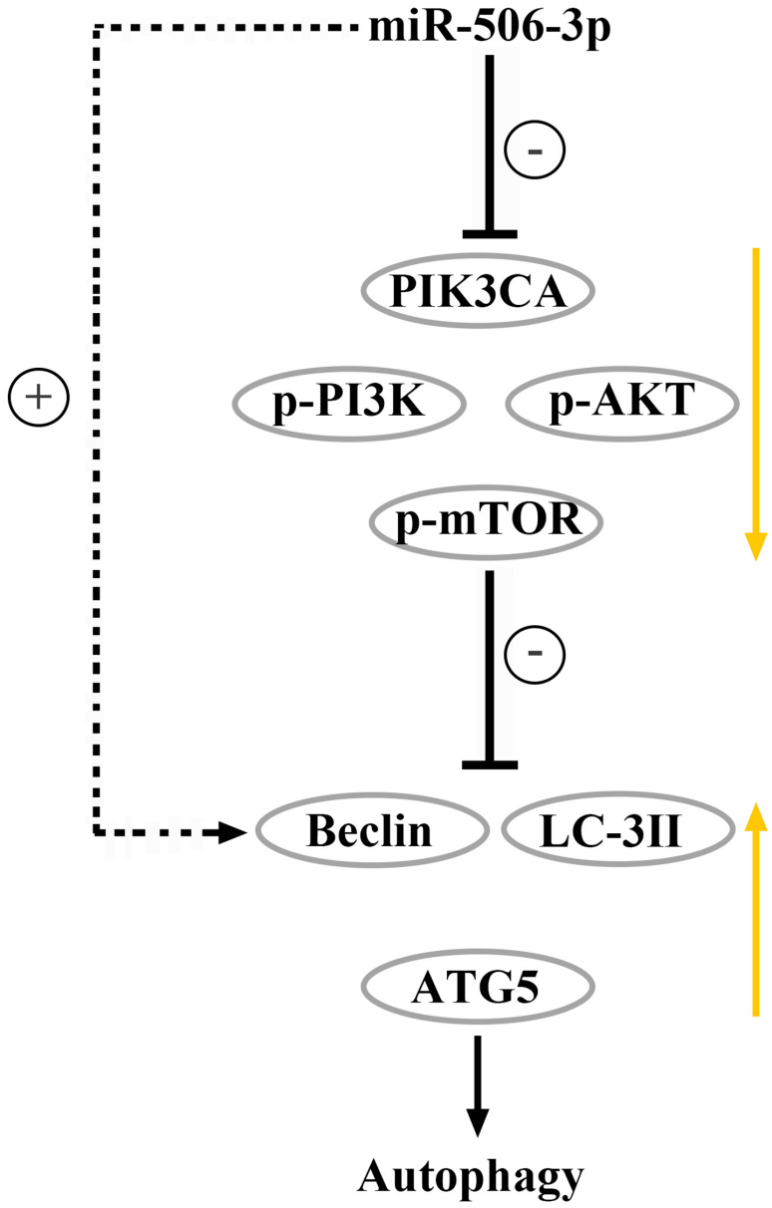
Overexpression of miR-506-3p could suppress the PI3K-AKT pathway via degrading PIK3CA, thereby enhancing autophagy of renal tubular epithelial cells in sepsis.

It was found by transmission electron microscopy that the number of mature mitochondria and autophagosomes in the miR-506-3p OE group was significantly increased compared with that in the NC group, while it was significantly reduced in the miR-506-3p KD group, indicating that miR-506-3p can increase the number of mitochondria and autophagosomes in kidney tissues, thereby enhancing autophagy of cells in kidney tissues (Figure 6A–6D). Therefore, based on the above results, it can be inferred that overexpression of miR-506-3p can inhibit the PI3K-AKT pathway by degrading PIK3CA, thereby enhancing autophagy of renal tubular epithelial cells in sepsis (Figure 7).

## DISCUSSION

Sepsis, one of the common complications of clinical acute and critical diseases [9], is primarily caused by infection with pathogens, such as bacteria, viruses, rickettsia, spirochetes, fungi and parasites [10], which can lead to multiple organ dysfunction in severe cases [11], accounting for 40–60% of complications of sepsis [12]. There is a study showing that LPS induces the enhanced autophagy of mouse renal tubular cells [13]. Autophagy exerts a protective effect in SA-AKI [14], which covers the autophagy signal transduction, formation of phagosomes, movement and deformation of phagosomes, formation of autophagosomes, fusion of autophagosomes with endosomes or lysosomes, formation of autolysosomes, and degradation of targets [15]. miRNAs are important regulators in many of the above links [16]. Recent studies have shown that miRNAs play an important role in the occurrence and development of SA-AKI [17]. For example, Cao LN et al. found that miR-21 can predict the occurrence of SA-AKI after cardiac intervention [18]. Deng X et al. found that miR-21 has an abnormal expression in the kidney of SA-AKI mice [19].

In the present study, it was found by bioinformatics analysis that PIK3CA was lowly expressed in SA-AKI. PIK3CA encodes the catalytic subunit p110 of class I PI3K, which activates PI3K through different mechanisms of interaction with the PI3K regulatory subunit p85 and with RAS-GTP [20]. Furthermore, KEGG pathway enrichment analysis using R-Studio showed that miR-506-3p had a targeted regulatory effect on PIK3CA, a key gene in the PI3K-Akt signaling pathway. Studies have shown that the expression of miR-506-3p in SA-AKI is opposite to the protein expression of the PI3K-Akt signaling pathway. The PI3K-Akt signaling pathway is an important pathway for autophagy, and PI3K and Beclin-1 are commonly-used indexes [21], which are highly important in autophagy. PI3K is ubiquitous in the cytoplasm in the resting state, and autophagy will be activated when p-PI3K is inhibited and the PI3K-Akt signaling pathway is blocked. Beclin-1 is a specific gene of autophagy in mammalians and a marker for autophagy, which is indispensable for the formation of autophagosomal precursors and autophagosomes [22].

The results of *in vivo* and *in vitro* experiments manifested that overexpression of miR-506-3p decreased the expressions of PIK3CA, p-PI3K and p-mTOR, but increased the expressions of Beclin, LC-3II and ATG5. After 740Y-P was added, the expressions of p-PI3K, p-mTOR, Beclin, LC-3II and ATG5 had no significant changes, but the expression of PIK3CA declined in miR-506-3p OE+740Y-P group compared with those in miR-506-3p NC+740Y-P group (*p* < 0.01). The above results demonstrate that exogenous overexpression of miR-506-3p can regulate the expression of autophagy-related proteins through inhibiting the PI3K pathway, thereby enhancing the autophagy of renal tubular epithelial cells in sepsis. Besides, highly expressed miR-506-3p in model group could alleviate kidney tissue damage, mitochondrial morphological changes and apoptosis, and increase the number of autophagosomes in renal tubular epithelial cells. It can be seen that miR-506-3p may enhance autophagy of renal tubular epithelial cells. The above findings indicate that overexpression of miR-506-3p in normal cells has no impact on the PI3K pathway, but overexpression of miR-506-3p in SA-AKI model can activate autophagy-related proteins through inhibiting the PI3K pathway, thereby enhancing autophagy of renal tubular epithelial cells in sepsis.

In conclusion, overexpression of miR-506-3p can suppress the PI3K-AKT pathway via degrading PIK3CA, thereby enhancing autophagy of renal tubular epithelial cells in sepsis, whose mechanism is related to the PI3K-AKT signaling pathway and autophagy protein Beclin.
